# *Modus operandi* of ClC-K2 Cl^−^ Channel in the Collecting Duct Intercalated Cells

**DOI:** 10.3390/biom13010177

**Published:** 2023-01-14

**Authors:** Anna Stavniichuk, Kyrylo Pyrshev, Viktor N. Tomilin, Mariya Kordysh, Oleg Zaika, Oleh Pochynyuk

**Affiliations:** Department of Integrative Biology and Pharmacology, The University of Texas Health Science Center at Houston, Houston, TX 77030, USA

**Keywords:** Cl^−^ intake, transcellular Cl^−^ reabsorption, Bartters’ syndrome type 3, pendrin, AE1, acid-base transport

## Abstract

The renal collecting duct is known to play a critical role in many physiological processes, including systemic water–electrolyte homeostasis, acid–base balance, and the salt sensitivity of blood pressure. ClC-K2 (ClC-Kb in humans) is a Cl^−^-permeable channel expressed on the basolateral membrane of several segments of the renal tubule, including the collecting duct intercalated cells. ClC-Kb mutations are causative for Bartters’ syndrome type 3 manifested as hypotension, urinary salt wasting, and metabolic alkalosis. However, little is known about the significance of the channel in the collecting duct with respect to the normal physiology and pathology of Bartters’ syndrome. In this review, we summarize the available experimental evidence about the signaling determinants of ClC-K2 function and the regulation by systemic and local factors as well as critically discuss the recent advances in understanding the collecting-duct-specific roles of ClC-K2 in adaptations to changes in dietary Cl^−^ intake and maintaining systemic acid–base homeostasis.

## 1. Mosaic Architecture of the Collecting Duct 

**Figure 1:**
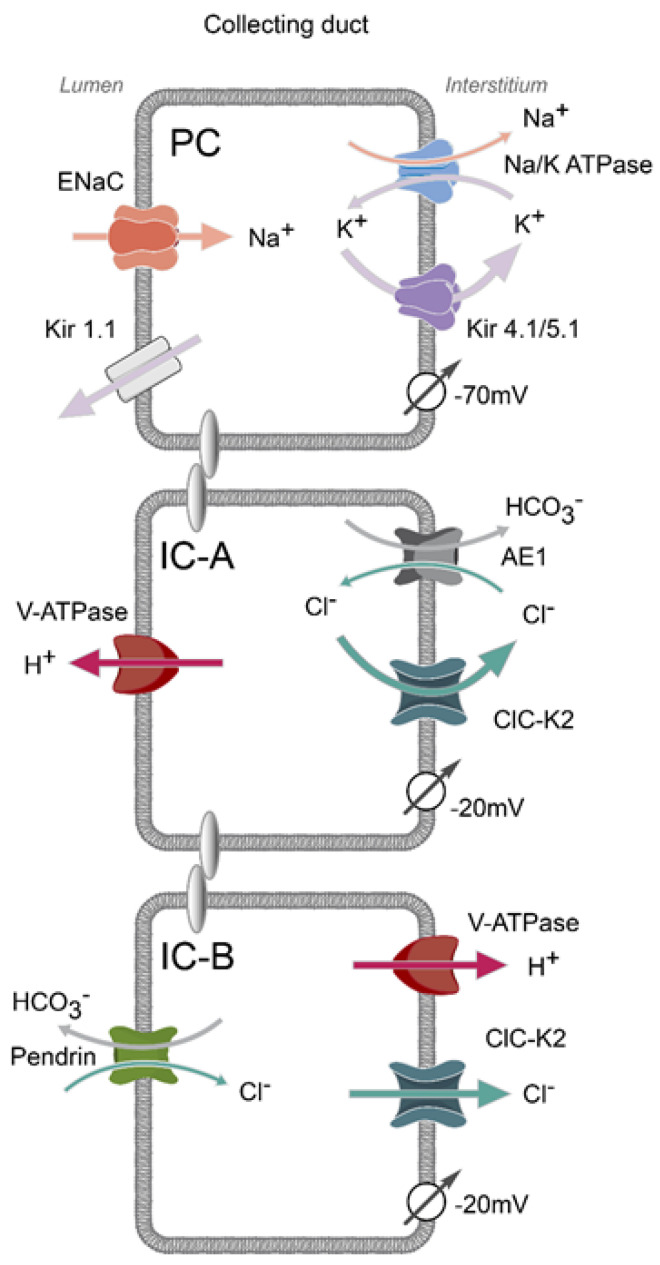
**Morphological aspects of the collecting duct.** The most abundant (2/3 of the total cell population) principal cells (PC) express the epithelial Na^+^ channel (ENaC) and potassium ROMK (K_ir_1.1) channel on the apical membrane and Na^+^/K^+^ ATPase and K_ir_4.1/5.1 potassium channel on the basolateral membrane (interstitial side). Due to predominantly K^+^ conductance, the basolateral membrane is hyperpolarized (−70 mV). Acid-secreting (A-type) intercalated cells express H^+^-ATPase on the apical side and anion exchanger type 1 (AE1) on the basolateral side. Base-secreting (B-type) intercalated cells express anion exchanger pendrin and H^+^-ATPase on the apical and basolateral membranes, respectively. ClC-K2 Cl^−^ channel is expressed on the basolateral membrane of both A- and B-types of intercalated cells. Intercalated cells have largely C^−^-selective electrogenic conductance and resting membrane voltage of −20 mV.

In fancy terms, the collecting duct system is viewed as the most distal guard post in controlling the systemic water–electrolyte balance to maintain whole-body homeostasis and to set chronic blood pressure. Indeed, several clinically notable pathologies, including nephrogenic diabetes insipidus, distal renal tubule acidosis type 1, pseudohypoaldosteronism (PHA) type 1, Liddle syndrome, etc., arise from genetic defects in the collecting duct transport systems [1]. The collecting duct is arguably the most complex and dynamically regulated segment of the renal nephron, in part due to the presence of two morphologically and functionally distinct cell types: principal and intercalated [2,3,4] (Figure 1). Moreover, intercalated cells can be subdivided into acid-secreting A-type, base-secreting B-type, and a transition phenotype non-A non-B most prominently observed in the connecting tubule and cortical collecting duct [3,4,5]. Strikingly, principal and intercalated cells are not electrically coupled and exhibit unique sets of transporting systems and physiological functions [5]. Thus, the prevalent principal cells (approximately 2/3 of the total population) possess predominantly cation conductance, thereby being involved in Na^+^ reabsorption and K^+^ secretion via the apically localized epithelial Na^+^ channel (ENaC) and renal outer medullary K^+^ (ROMK or K_ir_1.1) channels, respectively [2]. In contrast, intercalated cells “specialize” in the movement of anions, namely Cl^−^ and HCO_3_^−^, to regulate the acid–base balance and transcellular Cl^−^ reabsorption with initial entry mostly via the apical Cl^−^/HCO_3_- exchanger, pendrin (*Slc26A4*) [3,4]. Although it is commonly believed that appreciable Cl^−^ movement occurs in a paracellular manner in the collecting ducts (~30% for rabbits [6]), the tight junctions are only marginally more selective for Cl^−^ versus Na^+^ (ratio is 1.2–1.3:1) [7], indicating rather passive concomitant NaCl flux, which similarly occurs in upstream tubular segments, such as thick ascending limbs [8].

Abundant basic and clinical evidence demonstrates a significant role of the collecting duct in establishing a salt-sensitive pattern of blood pressure in different ethnic groups of patients [9,10,11]. Indeed, the pressor effects of an elevated dietary Na^+^ intake and augmented ENaC-mediated Na^+^ reabsorption received extensive focus and investigation [12,13,14,15,16]. However, emerging evidence suggests that Cl^−^ homeostasis is equally as important for blood pressure control [17]. For instance, Dahl salt-sensitive or stroke-prone spontaneously hypertensive rats became hypertensive when fed a high NaCl diet but not high Na^+^ bicarbonate or other Cl^−^ substitutes-based diets [18,19,20]. While it is commonly perceived that salt consumption is the primary source of Na^+^ and Cl^−^ intake, their concentrations do not go hand-in-hand in processed foods [17], suggesting the necessity to independently regulate Na^+^ and Cl^−^ homeostasis. In this regard, it is conceivable to assume that such a “yin–yang” architecture of the collecting duct with principal cells controlling Na^+^ and intercalated cells mediating Cl^−^ reabsorption is perfectly suited to accomplish this task.

Originally, the majority of the research effort was devoted to uncovering the physiological relevance of the major apical transporting systems, such as ENaC and pendrin, in principal and intercalated cells, respectively, by systemic hormones (aldosterone and Ang II) and local paracrine factors, such as bradykinin signaling [21,22]. In contrast, the significance of the basolateral membrane in these cells remained largely underappreciated. At the same time, the recent breakthrough studies demonstrated the central role of the basolateral conductance in controlling the apical NaCl entry in the upstream distal convoluted tubule [23]. Here, the activity of the K_ir_4.1/5.1 potassium channels sets the basolateral membrane voltage to drive exit of Cl^−^ via the ClC-K2 channel. This, in turn, determines intracellular Cl^−^ levels and the functional status of the Cl^−^-sensitive with-no-lysine (K) kinases, WNK1 and WNK4, the major regulators of the apical NaCl co-transporter, NCC [24]. WNK inhibition causes rapid de-phosphorylation of the cotransporter within minutes after a dietary K^+^ load, thus shifting the Na^+^ reabsorption to the downstream collecting duct to perform the Na^+^/K^+^ exchange favoring urinary K^+^ elimination [25]. Interestingly, the K_ir_4.1/5.1 and ClC-K2 channels are also major determinants of the basolateral conductance in the collecting duct. However, there is one significant difference, i.e., K_ir_4.1/5.1 is restricted to principal cells, and ClC-K2 is present only in intercalated cells [26,27,28] (Figure 1). Such a striking change in the expression patterns argues for the site-specific roles of the cation- and anion-selective basolateral conductance in the different cell types of the collecting duct. The roles of basolateral K^+^ conductance in the principal cells have been reviewed recently [29,30]. Here, we critically discuss the recent findings regarding the signaling mechanisms and novel proposed roles of the Cl^−^ conductance with its major contributor, the ClC-K2 channel in intercalated cells, with respect to Cl^−^ reabsorption and adaptation to systemic acid–base stimuli.

## 2. Basolateral Electrogenic Cl^−^ Conductance Is Mediated by ClC-K2 in Intercalated Cells

Direct electrophysiological studies in isolated collecting ducts found an uncharacteristically low basolateral membrane voltage of approximately −20 mV in both A- and B-types of intercalated cells as opposed to a very hyperpolarized voltage of −70 mV in principal cells and in the epithelial cells of upstream distal convoluted tubule and thick ascending limb segments [25,28,31,32]. This is consistent with predominant Cl^−^ conductance considering Nernst’s equilibrium potential for the physiological distribution of Cl^−^ ions with no significant contribution of K^+^. Consistently, intercalated cells are the only cell type with no notable expression of the Na^+^/K^+^ ATPase pump on the basolateral membrane in the renal nephron, with transmembrane transport being energized by the V-ATPase proton pump expressed on the apical and basolateral membranes of the A- and B-types, respectively [4,31]. Patch clamp studies revealed anion-selective 10 pS conductance in intercalated cells [26,28,33], whereas a 40 pS K^+^-selective channel was found on the basolateral membrane of principal cells [27,28,34], with both channels being simultaneously present only in a few cells of the connecting tubule, potentially representing the transition segment, but never in the collecting duct [27]. The following studies demonstrated that the deletion of ClC-K2 abolishes the identical 10 pS anion-selective conductance on the basolateral membrane of the distal convoluted tubule cells [35], thus providing convincing evidence in support of the molecular identity of Cl^−^ conductance in intercalated cells of the collecting duct. Consistently, it has recently been shown that the intercalated cell-specific deletion of the ClC-K2 channel abolishes over 90% of Cl^−^ dependent intracellular pH (pH_i_) changes in both the A- and B-types [5].

It should be noted that Cl^−^ conducting pathways other than ClC-K2 are also present on the basolateral membrane of intercalated cells. This includes electroneutral K^+^-Cl^−^ co-transporters KCC4 and KCC3a [36,37,38,39]. In the collecting duct, both cotransporters are predominantly located in intercalated cells and not in principal cells. However, there is no evidence that KCCs participate in transcellular Cl^−^ reabsorption. Thus, no apparent urinary electrolyte wasting was reported in KCC4 knockout mice [37]. Quite surprisingly, KCC3a-/- mice are hypertensive, but this phenotype has a neurogenic origin due to increased sympathetic tone [40]. In general, KCCs are known to be activated by hypotonicity and play a critical role in the regulatory volume decrease [41]. In addition, a LRRC8E subunit of volume-regulated anion channel (VRAC) has been found specifically in intercalated cells [42]. Altogether, this might be particularly important for maintaining the intracellular homeostasis of intercalated cells (which have low water permeability) during notable osmotic gradients existing in the collecting duct. Until now, there have been no studies testing this hypothesis. Interestingly, KCC4 knockout in mice leads to renal tubular acidosis, pointing to dysfunctional A-type of intercalated cells [37], whereas KCC3a might play a role in the adaptation to metabolic alkalosis, indicating its role in the B-type [38]. The molecular mechanisms of the activation of the KCCs by systemic acid–base stimuli are largely enigmatic.

## 3. Structural and Biophysical Properties of ClC-K2 Channel

While commonly referred to as the Cl^−^ channel, ClC-K2 is capable of conducting various anions with a permeability profile of Cl^−^>Br^−^>NO_3_^−^>I^−^ [43,44,45]. It belongs to the ClC family of anion channels/transporters with a common structure of two identical subunits, each having 12 α helixes (A-L) traversing the plasma membrane [46]. A functional channel has two “hourglass”-shaped permeation pores, which could operate independently in some ClC channels, leading to the characteristic two-step openings controlled by fast gates (located in each subunit) and a common slow gate (responsible for coordinated opening of both subunits), as could be observed with patch clamp electrophysiology [47]. Interestingly, ClC-K2 exhibits virtually exclusively one-step slow openings in the range of one hundred millisecond-to-seconds, indicative of the coordinated concomitant opening of both pores [26,48,49]. It is likely attributed to the lack of a critical glutamate at the vicinity of the conducting pore (substituted to neutral valine in ClC-K2), which is thought to be essential for controlling the “fast gate” and conferring voltage dependence [47,50]. Consistently, ClC-K2 does not demonstrate apparent changes in open probability of the channel at different membrane voltages [26,33,43,48,49,51]. Instead, ClC-K2 activity can be potentiated by extracellular Ca^2+^ via direct binding to the extracellular amino acids E261 and D278, whereas H^+^ (low pH) blocks ClC-K2 by binding to potentially the same region [52]. Moreover, a recent cryo-EM structure of a bovine ClC-K channel revealed a marked widening of the ion-permeation pathway due to the displacement of an adjacent αC-D helix, which partially protrudes into the conducting pore in other members of the ClC family [46]. This narrowing is proposed to underlie the puzzling observation that many ClCs could operate as both Cl^−^-permeable channels as well 2Cl^−^/H^+^ exchangers. This mode does not seem to be applicable to ClC-K2 [53]. Another unique attribute of ClC-K2 is its auxiliary β subunit, barttin [44]. Barttin is a small two-transmembrane domain protein, which is critical for ClC-K2 trafficking to the plasma membrane [54]. The absence of barttin causes ClC-K2 retention in the Golgi [55]. The ClC-K2-barttin interaction seems to require the palmitoylation of the latter for the proper plasma membrane insertion of the complexes [56]. However, barttin does not seem to participate in the conductance of the pore, since the reconstitution of purified bovine ClC-K into lipid vesicles leads to a robust Cl^−^ influx in the absence of barttin [46].

## 4. Expression Sites and Physiological Relevance of ClC-K2

Unlike the broad tissue distribution of ClC family members, ClC-K2 and its nearly twin sibling, ClC-K1 (has 90% identical amino acid sequence with ClC-K2), have very restricted expression patterns, which are reflected by the index “K” or kidney, the place where both channels were cloned [57,58]. The inner-ear epithelium, and more specifically the K^+^ secreting marginal cells of the *stria vascularis*, is the only significant extra-renal site of simultaneous ClC-K2 and ClC-K1 expression [44,59]. Interestingly, ClC-K2 and ClC-K1 show almost no overlap in adult kidneys. Thus, ClC-K2 is expressed on the basolateral membrane of the thick ascending limb, distal convoluted tubule, and AQP2-negative (intercalated) cells of the collecting duct [60]; whereas ClC-K1 is found on the basolateral and also apical plasma membranes of the thin ascending limb in the inner medulla [61,62]. As of now, there are no reported diseases associated with mutations in *CLCNKA* encoding ClC-Ka (orthologue of ClC-K1) in humans. By contrast, over 20 different mutations in *CLCNKB* encoding ClC-Kb (orthologue of ClC-K2) have been found to be responsible for Bartters’ syndrome type 3 (reviewed in [63]). Bartters’ syndrome is an autosomal recessive tubulopathy, which is manifested as hypokalemic metabolic alkalosis, polyuria, hypotension, and secondary hyperaldosteronism [64,65]. Hypocalcemia with nephrocalcinosis and hypomagnesemia can also be present in patients with Bartters’ syndrome [66,67]. Consistently, ClC-K2 deletion in mice faithfully recapitulated the major features of Bartters’ syndrome: renal salt loss, hypotension, hypokalemia, and metabolic alkalosis [35,68]. While, originally, Bartters’ syndrome was largely attributable to the disruption of NaCl reabsorption in the thick ascending limb and resistance to loop diuretic furosemide [64,69], animal knockout models also exhibited a dramatic reduction of salt reabsorption in the distal convoluted tubule and resistance to thiazide diuretics [35,68]. Thus, the overall conclusion was that ClC-K2/ClC-Kb dysfunction impairs basolateral Cl^−^ exit in the thick ascending limb and distal convoluted tubule to compromise the activity of the apical sodium-chloride co-transporters NKCC2 and NCC, respectively. At the same time, the potential contribution of ClC-K2 deletion in the collecting duct for the overall salt wasting phenotype and electrolyte imbalance was not elucidated in these animal models. 

In contrast to ClC-K2, the deletion of ClC-K1 did not cause a Bartters’-like pathology in mice. Instead, CLC-K1-/- mice developed nephrogenic diabetes insipidus due to compromised urinary concentrating ability and diminished hypertonic interstitial osmolarity in the inner medulla [62], which is consistent with the thin ascending limb being the major site of ClC-K1 expression [61,62]. Interestingly, concomitant ClC-K1 and ClC-K2 deletion leads to a more pronounced urinary salt wasting phenotype than that observed in ClC-K2 knockouts. Double knockouts failed to thrive, with there being 100% mortality by 3 weeks, which cannot be rescued by exogenous saline administration [70]. Consistently, Bartter’s syndrome type 4, due to mutations in the *BSND* gene encoding barttin, exhibits a more detrimental phenotype than Bartter’s syndrome type 3 [71]. It likely reflects the significance of barttin in promoting the translocation of both ClC-Kb and ClC-Ka to the plasma membrane. Recent evidence suggests that ClC-K1 and ClC-K2 are much more co-localized in the inner and outer medulla in embryonic and early neonatal kidneys [70]. It is suggested that thick ascending limb development and medulla maturation lead to a gradual dissociation in the expression patterns, with ClC-K2 being more abundant in the outer medulla and cortex, whereas ClC-K1 remains in the inner portion [70]. It was further proposed that the lack of ClC-K2 could be partially compensated by an upregulation of ClC-K1 in the ascending limb, thus explaining the less-severe phenotypes of patients with Bartter syndrome type 3 (classic) compared to antenatal types 1 and 2 caused by the dysfunction of the apical NKCC1 and ROMK, respectively [64,65,69]. In contrast, ClC-K2 knockout mice have markedly diminished responses to loop diuretic furosemide [35,68], thus arguing against the notable compensatory role of ClC-K1. Moreover, early stage ClC-K2 deletion led to impaired renal medulla and the thick ascending limb development [70], whereas no gross abnormalities in kidney structure were reported in other ClC-K2 knockouts [35,68]. The nature of these discrepancies is unclear.

## 5. Regulation of ClC-K2 in the Collecting Duct by Systemic and Local Factors

**Figure 2:**
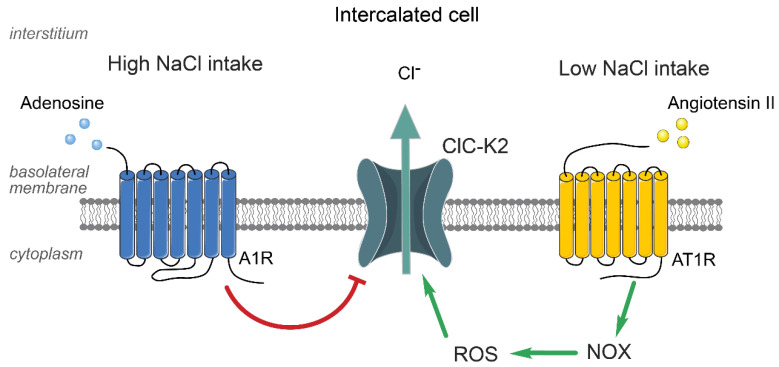
**Molecular determinants of ClC-K2 regulation in intercalated cells during variations in dietary Cl^−^ intake.** Elevations in Angiotensin II levels during hypovolemia (low NaCl intake) increase ClC-K2 open probability (P_o_) and expression by activating Angiotensin receptors type 1 (AT1R) with subsequent activation of NADPH oxidase (NOX) and reactive oxygen species (ROS) generation. In contrast, high NaCl intake leads to increased production of interstitial adenosine levels to decrease ClC-K2 activity via adenosine type 1 receptors (A1R).

While there is a general consensus that ClC-K2 is an essential component of the reabsorptive machinery of the renal nephron, the available experimental and clinical evidence focuses exclusively on its function in the thick ascending limb and the distal convoluted tubule but not in the collecting duct. This inequity could partially arise from the notion that hypotension and urinary salt wasting in Bartter’s syndrome type 3 are commonly accompanied by disturbances in the homeostasis of divalent cations, Ca^2+^ and Mg^2+^, which are reabsorbed upstream of the collecting duct [64,69,72,73]. At the same time, patch clamp studies show a comparably functional ClC-K2 expression on the basolateral membrane of the distal convoluted tubule and the intercalated cells of the collecting duct [26,33,49]. Considering the well-established role of the collecting duct in maintaining the circulating volume during variations in dietary salt intake, it is conceivable to propose that ClC-K2 is well-suited to controlling transcellular Cl^−^ reabsorption by intercalated cells. Indeed, functional studies have shown that ClC-K2 activity and expression is inversely related to the amount of Cl^−^ in a diet [28]. Such regulation indicates a potential stimulatory input from the Renin-Angiotensin Aldosterone System (RAAS). Indeed, physiologically relevant nanomolar Ang II levels [74] acutely increased the macroscopic Cl^−^ conductance and ClC-K2 open probability in freshly isolated collecting ducts by acting on AT1 receptors (AT1R) [75]. In addition, renal ClC-K2 levels were 50% reduced in mice lacking AT1R, also suggesting a permissive role of Ang II in setting ClC-K2 expression [75]. Pharmacological studies found that the stimulation of AT1R leads to the activation of NADPH oxidases (NOX) to increase the generation of reactive oxygen species (ROS) in the collecting duct cells. A schematic presentation of the stimulatory effect on Ang II on ClC-K2 is shown in Figure 2. Of note, the time course of Ang II-induced ROS production is similar to the time course of the increases in the ClC-K2 open probability, indicating the direct effect of ROS on the channel [75]. The exact molecular mechanism and domain/motif within the channel have yet to be elucidated. 

**Figure 3:**
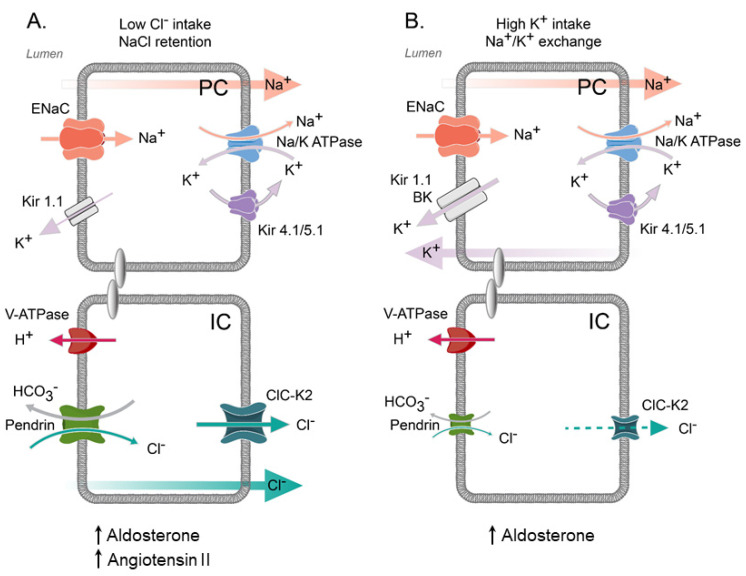
**Separation of cation and anion fluxes by principal and intercalated cells.** (**A**) Dietary NaCl restriction (low Cl^−^ intake) increases ENaC activity in principal cells by aldosterone and Angiotensin II. Ang II increases ClC-K2 and pendrin to promote transcellular Cl^−^ reabsorption by intercalated cells. Concomitant Na^+^ and Cl^−^ reabsorption diminishes the driving force for K^+^ secretion by principal cells, thereby precluding urinary K^+^ wasting. (**B**) High K^+^ intake increases ENaC activity in principal cells by aldosterone, whereas ClC-K2 activity is not augmented. In this case, electrogenic ENaC-dependent Na^+^ reabsorption promotes apical K^+^ exit in principal cells via ROMK and also large conductance BK channels. This Na^+^/K^+^ exchange facilitates urinary kaliuresis to eliminate the excess of dietary K^+^. The size of respective icons of transporting systems reflects their activity.

It should be noted that Ang II also increases ENaC activity in principal cells via the same intermediate downstream signaling [76]. Thus, it is conceivable to propose that elevations in Ang II levels during dietary salt restrictions increase NaCl reabsorption in the collecting duct by stimulating ENaC-mediated Na^+^ reabsorption by the principal cells and ClC-K2-dependent Cl^−^ transport by the intercalated cells to protect the circulating volume. The apical Cl^−^ entry most likely occurs via pendrin, which is also known to be upregulated by dietary Cl^−^ restriction and Ang II in B- and non-A-non-B types of intercalated cells [77,78,79]. Interestingly, the upregulation of transcellular Cl^−^ transport in this case occurs even in the absence of concomitant reduction of dietary Na^+^, suggesting an independent mode of operation [78]. Furthermore, the inhibition of ENaC with amiloride does not prevent transcellular Cl^−^ transport in perfused collecting ducts [79], and this can occur with minimal changes in tubular pH, suggesting that both A- and B-types of intercalated cells contribute to Cl^−^ reabsorption [80]. On the other hand, an increase in dietary K^+^ without concomitant elevation in Cl^−^ stimulated both apical and basolateral conductance in the principal cells but had no effect on ClC-K2 activity in intercalated cells [28]. It appears that such independent regulation of the conductance of principal and intercalated cells by dietary cations and anions, respectively, is also instrumental in controlling K^+^ secretion via the apical ROMK and large conductance BK potassium channels. During hypovolemia, the activation of electrogenic Na^+^ reabsorption via ENaC would be counterbalanced by the augmented Cl^−^ transport by intercalated cells. This reduces the driving force for K^+^ secretion, with K^+^ being recycled on the basolateral membrane via K_ir_4.1/5.1. By contrast, hyperkalemia does not lead to the stimulation of Cl^−^ conductance in intercalated cells, which enables maximal employment of the Na^+^/K^+^ exchange at the apical membrane of principal cells (Figure 3). This can, at least partially, explain the so-called “aldosterone paradox”, where comparable elevations in circulating aldosterone by the dietary K^+^ load or during hypovolemia lead to discrete patterns of urinary excretion, i.e., kaliuresis with no volume retention and volume retention with little or no K^+^ wasting [81]. Recall that aldosterone is the critical activator of ENaC activity and expression in the collecting duct [82,83]. By contrast, ClC-K2 does not seem to be stimulated by aldosterone in intercalated cells, since neither high K^+^ intake nor the mineralocorticoid receptor (MR) agonist deoxycorticosterone (DOC) affect basolateral Cl^−^ current and ClC-K2 single-channel activity [28]. Consistently, MRs in intercalated cells are usually phosphorylated at the position (S843-P), which precludes their activation and translocation to the nucleus [84]. Of note, S843-P phosphorylation of MR was further augmented by a high K^+^ intake [84]. Overall, concomitant elevations (Ang II) and aldosterone during hypervolemia stimulate both ENaC and ClC-K2 to favor NaCl reabsorption in the collecting duct, whereas a high K^+^ intake does not affect the ClC-K2 function, thus maximizing the driving force for K^+^ secretion. Of interest, the same mechanism could be also used by other hormones with notable actions on systemic electrolyte homeostasis. For instance, it was previously shown that insulin, known for its pro-kaliuretic actions [85,86], stimulates ENaC activity [87,88] but blocks ClC-K2 open probability in isolated collecting ducts [33]. In contrast, insulin growth factor-1 (IGF-1), which can induce ENaC-dependent hypertension [89,90], stimulates ClC-K2 activity and Cl^−^ conductance in intercalated cells [33].

Elevated dietary Cl^−^ intake decreases ClC-K2 activity and macroscopic Cl^−^ conductance in intercalated cells regardless of the accompanying cation [28]. While it is likely that the diminished Ang II levels might be responsible, other complementary cascades could also contribute to the inhibition of ClC-K2 during this condition. Thus, it was reported that chronic dietary salt loading greatly increases interstitial adenosine levels in the renal parenchyma [91]. Importantly, adenosine, by acting on A1 receptors, was capable of decreasing ClC-K2 activity in intercalated cells in a dose-dependent manner [92] (Figure 2). The physiological significance of this mechanism for regulation of ClC-K2 activity by dietary Cl^−^ intake was not explored.

Despite the aforementioned significant advances in uncovering modes of operation and signaling pathways to control ClC-K2 activity and expression in intercalated cells during variations in dietary Cl^−^ intake, it is not known whether the collecting duct ClC-K2-mediated Cl^−^ reabsorption contributes significantly to systemic homeostasis and blood pressure regulation. Distorted Na^+^ reabsorption in the principal cells, either due to loss-of-function or gain-of-function mutations in ENaC, leads to clinically relevant hypotensive PHA type 1 and hypertensive Liddle syndrome, respectively [93]. Pendrin deficient mice have blood pressures comparable with WT mice in unstressed conditions but become hypotensive and volume depleted when challenged with dietary NaCl deficiency [77]. Moreover, the overexpression of pendrin in intercalated cells leads to the development of salt-sensitive and more specifically Cl^−^-sensitive hypertension, even in the presence of downregulated ENaC levels [94]. Interestingly, gain-of-function polymorphism ClC-Kb^T481S^ in humans is also associated with the prevalence of salt-sensitive hypertension [95,96]. Thus, it is reasonable to anticipate that the renal phenotype of intercalated cell ablation/overexpression of ClC-K2 would be reminiscent of that of pendrin. Future studies contrasting the effects of ClC-K2 deletion in the renal nephron versus intercalated cells are necessary to support or disprove this hypothesis.

## 6. Contribution of ClC-K2 to H^+^ and HCO_3_^−^ Secretion by Intercalated Cells

**Figure 4:**
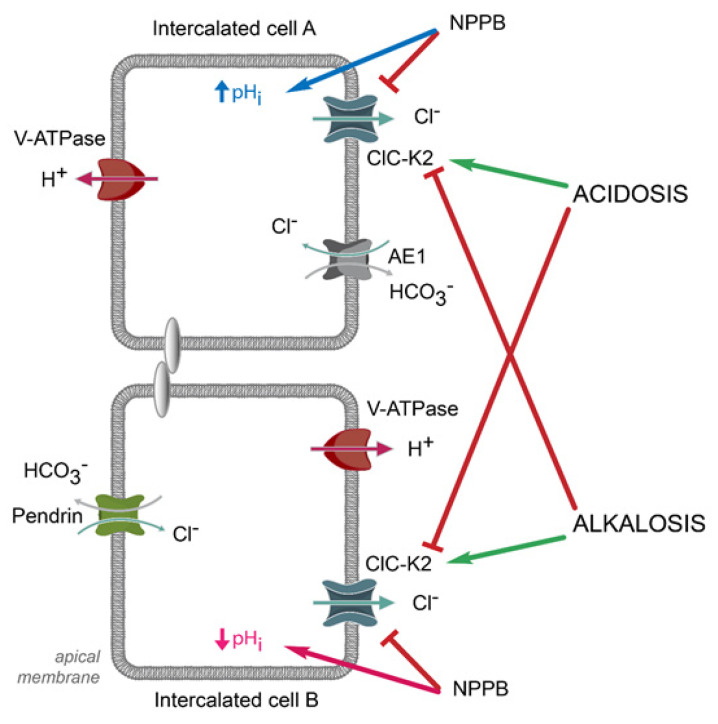
**ClC-K2 controls acid–base transport in intercalated cells.** Inhibition of ClC-K2 with 5-Nitro-2-(3-phenylpropylamino) benzoic acid (NPPB) compromises Cl^−^ dependent acid–base transport by intercalated cells leading to alkalization (increased pH_i_) in A-type and acidification (decreased pH_i_) in B-type. Systemic acid load (metabolic acidosis) increases ClC-K2 activity in A-type and decreases in B-type to facilitate urinary excretion of acids. Consistently, systemic alkali load (metabolic alkalosis) increases ClC-K2 activity in B-type and decreases in A-type to augment excretion of bases in urine.

The transport of acids and bases is the salient physiological aspect of intercalated cells in the collecting duct [4]. A- and B-types are virtually mirrored with respect to their function by moving H^+^ across the apical and basolateral membranes, respectively, via H^+^-ATPase [3,4]. This is accompanied by an anion/HCO_3_^−^ exchange on the opposite side, specifically via the anion exchanger type 1 (AE1, *Slc4a1*) on the basolateral membrane of A-type and pendrin on the apical side in of B-type [4]. Since Cl^−^ is the most prevalent anion, acid–base transport by intercalated cells is largely Cl^−^-dependent. Indeed, manipulations with extracellular Cl^−^ concentration elicit different changes in pH_i_, which is commonly used to discriminate between cell types [97,98]. In agreement, it was recently demonstrated that the inhibition of ClC-K2-mediated basolateral Cl^−^ efflux with NPPB caused intracellular alkalization in A-type but acidification in B-type [5] (Figure 4). While NPPB is known to have poor specificity for the channel, the selective deletion of ClC-K2 in intercalated cells decreased the NPPB-induced responses by more than 90%. Apart from serving as a valuable and reliable tool in discrimination of cell types in freshly isolated collecting duct preparations, ClC-K2 serves as a critical modulator of acid–base transport with ClC-K2 deletion causing over 50% decrease in H^+^ extrusion in A- and B-types [5]. Recall that Bartter’s syndrome type 3 due to ClC-Kb dysfunction is associated with metabolic alkalosis [63,99]. It is possible that this could be, at least partially, caused by the deficient HCO_3_^−^ secretion (i.e., alkali retention) of B-types of cells. Future studies with intercalated-cell-specific ClC-K2 deletion are necessary to test this prediction.

The presence of both A- and B-types of intercalated cells allows dynamic control of urinary pH in response to the systemic acid/base load. Indeed, perfused cortical collecting ducts can secrete H^+^ when animals are given ammonium water (acid load) and secrete HCO_3_^−^ when animals are loaded with alkali [100]. This was attributed not only to an increase activity/expression of either H^+^-ATPase or pendrin, but also to a relative increase in the percentage of A- or B- types in the collecting duct either due to the direct conversion of the polarity of the other type [97,101,102] or the further differentiation of a transitionary non-A non-B-type [103]. Functional studies found comparable ClC-K2 activity in A- and B-types of intercalated cells in freshly isolated cortical collecting ducts [5,28]. Interestingly, metabolic acidosis led to increased functional ClC-K2 activity in A-type and decreased ClC-K2 function in B-type cells [5] (Figure 4). Consistently, metabolic alkalosis induced the opposite changes. This implies that ClC-K2 is an important component of the collecting duct adaptation to systemic pH stimuli. Further studies are necessary to uncover the molecular details and physiological significance of such intricate regulation.

## 7. Final Remarks

Since cloning in early 2000s as the underlying cause of Bartters’ syndrome type 3 tubulopathy, ClC-Kb has been generally recognized as the major contributor to the basolateral Cl^−^ transport in the renal nephron essential for systemic electrolyte homeostasis and blood pressure control. Manipulation of the ClC-Kb function is very attractive from the pharmacological standpoint, due to the very restricted pattern of channel expression. At the same time, the fame (or infamy in the case of Bartter’s patients) was related to its function in the thick ascending limb and the distal convoluted tubule, whereas the roles of ClC-K2 in the collecting duct were generally overlooked. Luckily, we have gained a step in recent years by identifying collecting-duct-specific functions in the separation of cation and anion fluxes in principal and intercalated cells and in adaptation to changes in dietary Cl^−^ intake. We have also revealed critical upstream effectors, including Ang II and adenosine, to control ClC-K2 expression and activity in different physiological conditions. Moreover, ClC-K2 might be critical to control the function of A- and B-types of intercalated cells in adaption to systemic acid–base stimuli. However, we still miss an important translational component of how changes at the intercalated cell level affect the kidney function and overall cardiovascular health. We are looking forward to uncovering this exciting and promising chapter in the near future.
